# Adiposity, Biological Markers of Disease, and Insulin Resistance in Mexican American Adolescents, 2004-2005

**Published:** 2011-02-15

**Authors:** Anne R. Rentfro, Jeanette C. Nino, Cristina S. Barroso, Joseph B. McCormick, Susan P. Fisher-Hoch, Rosa M. Pones, Wendy Innis-Whitehouse, Mohammad H. Rahbar

**Affiliations:** University of Texas at Brownsville and Texas Southmost College, Nursing Department; The University of Texas at Brownsville, Brownsville, Texas; The University of Texas at Brownsville, Brownsville, Texas; The University of Texas at Brownsville, Brownsville, Texas; The University of Texas at Brownsville, Brownsville, Texas; Brownsville Independent School District, Brownsville, Texas; The University of Texas-Pan American, Edinburg, Texas; The University of Texas at Houston, Houston, Texas. Ms Nino is also affiliated with the Texas Southmost College

## Abstract

**Introduction:**

Rates of obesity and overweight, which frequently lead to type 2 diabetes, have increased dramatically among US children during the past 30 years. We analyzed associations between insulin resistance and other markers of disease in a sample of Mexican American adolescents from a severely disadvantaged community on the Texas-Mexico border.

**Methods:**

We analyzed results from 325 students from 1 high school in this descriptive study. We measured height, weight, waist circumference, blood pressure, blood glucose, and lipids; calculated body mass index; and estimated insulin resistance.

**Results:**

Approximately 50% of our sample (mean age, 16 y) were overweight or obese, and more participants were obese than overweight. More than 40% had high waist circumference, and 66% had elevated high-density lipoprotein cholesterol. These characteristics were already present in the youngest participants (aged 12 y). Although only 1% of participants had elevated fasting blood glucose, 27% exhibited insulin resistance and most of these were also obese. Similarly, participants with high waist circumference were more likely to exhibit insulin resistance than those with normal waist circumference.

**Conclusion:**

Participants in this sample had insulin resistance, a potent predictor of diabetes. Two markers, low high-density lipoprotein cholesterol and high waist circumference, were strongly linked to insulin resistance; the surrogate for central adiposity, waist circumference, exhibited strong association. We identified high levels of obesity and markers for future disease in our sample. These findings emphasize the need to address insulin resistance at least as early as adolescence to prevent adverse economic, social, and health consequences.

## Introduction

Rates of obesity and overweight in children and adolescents in the United States tripled from 1980 through 2002 and continued to increase into 2006 (1,2). Because obesity frequently progresses to type 2 diabetes, the burden of this disease will increase correspondingly (2-4). Hispanics are twice as likely as non-Hispanic whites to develop type 2 diabetes (5). Once considered rare, pediatric type 2 diabetes now accounts for almost one-third of all diabetes in children younger than 18 years (6), and Mexican American adolescents are disproportionately affected (7). Moreover, rates of type 2 diabetes are higher in southern border regions of Texas than in other areas of Texas or the United States, accounting for nearly half of all pediatric cases of diabetes in some areas of Texas (8).

Being overweight or obese, specifically having excess visceral adipose tissue, is associated with insulin resistance (IR), which is considered a precursor to type 2 diabetes (9). Genetic predisposition also contributes to IR, and Mexican Americans are at greater risk than non-Hispanic whites (10). Few standards exist for assessing risk among adolescents, but most experts agree that children who are obese and have metabolic abnormalities maintain these abnormal patterns into adulthood (4). Despite the recognized predisposition, little is known about predictors of type 2 diabetes, such as IR, in healthy Mexican American adolescents.

Research consistently links poor health outcomes to low socioeconomic status and poverty (11,12). Moreover, regardless of age or ethnicity, studies consistently link obesity to poverty (13,14). Goodman and colleagues (15) determined that IR was more pronounced in adolescents of low socioeconomic status compared with those of higher socioeconomic groups. More than 45% of children live below the federal poverty level along the Texas-Mexico border, compared with 31% in Texas and 28% in the United States (16,17). We established and analyzed metabolic, socioeconomic, and anthropometric characteristics in a sample of healthy Mexican American adolescents living on the Texas-Mexico border, in one of the poorest counties in the United States (18). 

## Methods

We performed a cross-sectional study of 325 adolescent students in a high school located in the southernmost part of the Texas-Mexico border, in Brownsville, Texas, a city with a population of 177,112 (16). We used a convenience sample because both the school and the consent process limited access to students.

We drew our sample from 9th- to 12th-grade students enrolled in 1 of 5 high schools in the school district. The 2,064 students in the study school make up 19% of the total district enrollment (11,020) (19) and represent the district socioeconomically and demographically. Students in this district's 5 high schools are mostly Mexican American (98% by parent self-report) (19); 99% are eligible for federally funded school meal programs (16,18,19).

The Committee for Protection of Human Subjects at University of Texas Health Science Center–Houston, the institutional review board for protection of human subjects at University of Texas at Brownsville and Texas Southmost College, and the school district board of directors approved the protocol. We obtained written consent from each participant and a parent or guardian before data collection.

### Recruitment

At mandatory school faculty meetings, we presented information about the study. Faculty members then invited study team members to their classrooms. Administrators permitted access only to elective courses containing health objectives (eg, child development, health occupations, Junior Reserve Officer Training Corps), which included less than half of the student body. In addition to giving presentations, the study team promoted the study through health fairs, information tables in high-traffic areas, flyers, and public address announcements.

Study personnel maintained a presence on campus during the data collection phase. Although school officials were supportive, they prohibited students from leaving class to participate. The school nurse and study coordinator instead scheduled appointments. Participants completed the study before school or during free time. The study coordinator and principal investigator collected data, including a fasting blood specimen, and height, weight, and waist circumference (WC) measurements. Participants rescheduled appointments if they arrived nonfasting. After we obtained fasting measures, participants received juice and crackers. The school nurse coordinated counseling and follow-up for abnormal anthropometric values.

The sample included students aged 15 to 18 years. Students who self-reported pregnancy or physical conditions influencing anthropometric measures, such as amputation, were excluded. Participants reported to the nurse's office for data collection. Data were collected prospectively on 325 participants (16% participation rate) Monday through Friday between February 2004 and March 2005, except in summer months when school was not in session.

### Measures

Students self-reported age, birth date, grade, and ethnicity. We categorized age as early adolescence (aged <15 y), middle adolescence (aged 15-17 y), and late adolescence (>17 y).

We used standard equipment and methods to measure weight and height. Participants removed their shoes and stood motionless on a portable electronic scale with feet slightly apart and weight equally distributed. We recorded height by using a portable stadiometer with participants standing tall, holding their breath, and looking straight ahead. We calculated body mass index (BMI) as weight in kilograms divided by height in meters squared (kg/m^2^). We classified participants with a BMI in the 85th percentile to less than 95th percentile by age and sex as *overweight* and those with a BMI in the 95th percentile or higher* as obese*, on the basis of Centers for Disease Control and Prevention growth charts (20).

We determined central adiposity by measuring WC as participants remained in a standing position, breathing normally. We used Fernandez and colleagues' (21) estimates of WC percentiles by age, sex, and height for Mexican American adolescents, which were derived from the National Health and Nutrition Examination Survey. We considered WC in the 75th percentile or higher for age, sex, and height as high (21).

We measured blood pressure according to the *Fourth Report on the Diagnosis, Evaluation, and Treatment of High Blood Pressure in Children and Adolescents* protocol (22). For analysis, we used the mean of 2 systolic values taken a few minutes apart by the same examiner. Using standards from the National High Blood Pressure Education Program that account for sex, age, and height, we considered systolic blood pressure in the 90th percentile or higher as high (22).

After participants fasted for 10 hours, we collected blood to measure fasting blood glucose, insulin, and lipids. We used standard equipment and methods to determine blood glucose and insulin levels. We estimated IR with the formula for homeostasis model assessment for insulin resistance (HOMA-IR) (23,24). We also used standard equipment to measure plasma lipids, including triglycerides, total cholesterol, high-density lipoprotein (HDL) cholesterol, and low-density lipoprotein (LDL) cholesterol.

We used the 2010 American Diabetes Association Standards of Medical Care to define fasting blood glucose values of at least 7.0 mmol/L as elevated, and values between 5.6 and 6.9 mmol/L as impaired fasting glucose (25). We defined IR as HOMA-IR of at least 3.16 (23). We considered triglyceride values of at least 11.1 mmol/L (logarithmically transformed for analysis) as high (26,27). We used cutpoints for HDL, LDL, and total cholesterol in adolescents recommended by the National Cholesterol Education Program Adult Treatment Panel for analyses (26,27).

### Analysis

No observations were eliminated because of missing values; however, where values for individual variables were missing, we specified denominators. For univariate analyses, separate logistic regression models used IR as the dependent variable; BMI, WC, age, sex, HDL cholesterol, LDL cholesterol, triglycerides, and total cholesterol were the independent variables. Multivariate logistic regression with IR as the dependent variable controlled for potential confounding and effect modification. We used SAS version 9.1.3 (SAS Institute, Inc, Cary, North Carolina) to perform the analyses.

## Results

### Participation

Of 2,064 enrolled students, 337 adolescents consented to participate; 325 completed 1 data collection examination appointment. We used standard protocols with reminder telephone calls and attempts to reschedule missed appointments. Missing values generally resulted from inability to obtain sufficient blood volume specimens or from incomplete examinations. Twelve participants failed to arrive for their appointments. After 5 attempts to reschedule, we withdrew those participants from the study.

Of the 325 adolescents, 65% (n = 211) were girls, 92% (n = 298) reported being of Mexican American descent, and 66% (n = 213) reported that all 4 grandparents were born in Mexico. Most participants (average age, 16 y) attended 9th (43%) or 10th (25%) grade compared with the district's enrollment for 9th and 10th grade of 37% and 28%, respectively.

Half of participants were categorized as overweight or obese. The largest proportion, 27%, were obese (boys 33%, girls 24%). More than one-third of participants exhibited WC measures indicative of central adiposity.

### Biomarkers of adverse health outcomes

Abnormal metabolic values occurred with regularity in these adolescents who might otherwise be considered healthy (Table 1). Obese participants were nearly 10 times more likely to exhibit IR than participants who were not obese (Table 2). Overweight participants were 2.7 times more likely to exhibit IR than those who were not overweight. Although significant, total cholesterol's association with IR was one of the weakest relationships noted at the univariate stage. We found that central adiposity consistently demonstrated close association with IR. Of participants with high WC, 53% exhibited IR, compared with 10% of participants with normal WC. Therefore, WC was strongly associated with IR and contributed to identifying adolescents in this sample who were most at risk for type 2 diabetes. We found no significant association between IR and sex, age, or fasting blood glucose.

On the basis of these findings, we tested IR as the outcome variable in a multivariate model with WC and HDL cholesterol (Table 2). Age, sex, and BMI remained in the model because of their associations with IR in other populations, even though BMI correlated with WC (r^2^= 0.60; *P* < .001). The multivariate model excluded total cholesterol, triglycerides, and LDL cholesterol because of weaker association in univariate analysis and because these lipid values covary inversely with HDL cholesterol. Because of the high number of participants with elevated HDL cholesterol (66%), which is considered modifiable with the use of nonpharmacologic physical activity interventions, this variable remained in the model. The association with IR remained nonsignificant for age and sex in the multivariate model. Participants with high WC measures and elevated HDL values were more likely to exhibit IR than those with normal values.

## Discussion

Although predisposed to diabetes, Mexican American adolescents have been underrepresented in studies exploring markers that predict IR to inform the science of preventing type 2 diabetes. Adolescents in our study were nearly twice as likely to be obese as Mexican American adolescents nationally (1). In fact, obese participants outnumbered overweight participants. The highest recorded BMI of 49.9 kg/m^2^ highlights the extreme nature of obesity in this sample. Furthermore, adolescent boys in our study were as likely to be overweight as adolescent girls and exhibited the same rate of IR (27%). However, boys in this sample had higher rates of obesity than girls. Lack of significant association between IR and fasting blood glucose in this sample may reflect the low rate of abnormal fasting blood glucose values. Transition to glucose intolerance in type 2 diabetes occurs gradually with chronic IR.

Adolescents in this sample exhibited obesity, IR, and metabolic abnormalities at rates usually associated with older adults. Experts recommend prevention programs and screening and lifestyle interventions for type 2 diabetes among children and adolescents (28,29). Adolescents most at risk for type 2 diabetes are likely to be among those who exhibit IR at an early age.

Keskin and colleagues' (23) cutpoint identified obese adolescents who exhibited IR. Use of top tertile and quartile cutpoints in our sample resulted in high IR, 33% and 25% respectively. Top tertile (2.92) and top quartile (3.36) HOMA-IR values were higher than Keskin's tertile and quartile results. These calculations provide further support for use of Keskin's cutpoint in adolescents. Multiple regression analyses that explore associations as IR rises may provide a more sensitive approach to exploring predictors with participants in the normal range for IR, rather than identifying associations among factors linked only to the group outside that range.

Although BMI for age and sex for both overweight and obese participants was significantly associated with IR in univariate analysis, significance disappeared in multivariate analysis. One possible explanation may be that BMI reflects an association when considered alone but is weakened in the context of more potent predictors. Other studies of adolescents provide support for using WC as an additional marker of risk for early metabolic disease (30-32). The strong association between low HDL cholesterol and IR also contributed to identification of participants who were most at risk for disease. Disease risk profiles that feature HDL cholesterol are less common in studies of adolescents than risk profiles featuring WC or LDL cholesterol (33,34). Our analyses provide support for the contribution of WC and HDL cholesterol to adolescent risk profiles.

Our study had several limitations. Adolescents present a particular challenge in nonclinical settings such as schools, where overnight fasting for venipuncture is involved. Boys made up 51% of the study school's enrollment; however, similar to other studies, half as many boys (35%) as girls (65%) participated. Data were collected from only 1 school of 5 in the district. Administrative requirements for students to participate outside of class time and being confined to health education classes limited enrollment and permitted access to adolescents who were more likely to be interested in healthy lifestyles, possibly resulting in an overrepresentation of more active, less obese adolescents and bias toward underreporting of obesity and related disorders. Lack of established cutpoints for Mexican American adolescents presented an additional limitation. The nonexperimental descriptive design that selected a specific school in 1 city limits generalizability of these findings. Nevertheless, overall our findings indicate the need for similar studies in other Mexican American adolescent populations and other ethnic groups, particularly those in unique locations.

This study explored associations among adiposity, biological markers of disease, and IR in a sample of Mexican American adolescents from one of the poorest school districts in the United States. Our findings offer additional support for the association of IR with obesity and markers of disease in Mexican American adolescents and suggest that continuing state and school policies are needed to address the obesity epidemic from a population-based perspective. Prospective interventional studies are needed to prevent obesity and obesity-related diseases in regions populated with Mexican Americans and in areas with high poverty.

## Figures and Tables

**Table 1:** Anthropometric Measures and Biomarkers in a Sample of Mexican American Adolescents, South Texas, 2004-2005

Characteristic	n^a^	Mean (SD)	Abnormal, No. (%)
Age, y	325	15.8 (1.4)	NA
Systolic BP, percentile for age, sex, and height	320	109 (10.5)	NC
Body mass index, kg/m^2^	321	25.8 (5.9)	NA
Body mass index, percentile for age and sex^b^	321	76.9 (24.3)	160 (50)
Waist circumference, percentile for age and sex^c^	321	85.7 (16.0)	132 (41)
Fasting blood glucose, mmol/L^d^	302	4.9 (0.5)	3 (1)
Impaired fasting glucose^e^	299	NA	25 (8)
Insulin, *µ*U/mL^2^	299	11.8 (8.6)	NA^f^
HOMA-IR^g^	297	2.6 (2.1)	82 (27)
HDL cholesterol, mmol/L^h^	302	0.1 (0.3)	200 (66)
LDL cholesterol, mmol/L^h^	302	2.3 (0.6)	91 (30)
Triglycerides, mmol/L^h^	239	0.9 (0.5)	59 (20)
Total cholesterol, mmol/L^h^	302	3.9 (0.7)	51 (20)

Abbreviations: SD, standard deviation; NA, not applicable; BP, blood pressure; NC, not calculated; HOMA-IR, homeostasis model assessment for insulin resistance; HDL, high-density lipoprotein; LDL, low-density lipoprotein.

a Sample size of <325 reflects unfinished examinations or inadequate blood volume for all tests. All complete test results were used regardless of whether the participant completed all tests.

b The normal value is <85th percentile (20).

c The normal value is <75th percentile (21).

d The normal value is <7.0 mmol/L (13).

e Defined as 5.6-6.9 mmol/L (13).

f No standardized normal values exist for insulin in adolescents.

g The normal value is <3.16 (5).

h The normal values are HDL cholesterol >1.2 mmol/L for adolescent boys aged 14-19 y and >1.3 mmol/L for all others; LDL cholesterol <2.8 mmol/L; triglycerides <1.2 mmol/L; and total cholesterol <4.4 mmol/L (26,27).

**Table 2:** Odds of Having Insulin Resistance, by Biologic Markers, Mexican American Adolescents, South Texas, 2004-2005

Characteristic	No. (% with IR)	OR (95% CI)Univariate	Adjusted OR (95% CI) Multivariate
**Sex**			
Boys	105 (27.6)	1 [Reference]	NA
Girls	192 (27.6)	1.0 (0.6-1.7)	NA
**Age category^a^**			
Early adolescence	59 (27.1)	1 [Reference]	NA
Middle adolescence	198 (26.3)	1.0 (0.5-1.8)	NA
Late adolescence	40 (35.0)	1.5 (0.6-3.4)	NA
**Body mass index^b^ percentile for age and sex**			
Normal	148 (12.2)	1 [Reference]	
≥85th to 95th (overweight)	67 (26.9)	2.7 (1.3-5.5)	1.0 (0.4-2.6)
≥95th (obese)	81 (56.8)	9.5 (4.9-18.4)	1.6 (0.6-4.5)
**Waist circumference percentile for age, sex, and height**			
Normal	179 (10.3)	1 [Reference]	
≥75th	117 (53.0)	9.7 (5.4-17.9)	6.9 (2.7-17.5)
**Triglycerides^c^**			
Normal	239 (19.7)	1 [Reference]	NA
Abnormal	58 (60.3)	6.2 (3.4-11.5)	NA
**HDL cholesterol^c^**			
Normal	98 (13.0)	1 [Reference]	
Abnormal	199 (35.0)	3.6 (1.9-6.9)	2.4 (1.2-5.1)
**LDL cholesterol^c^**			
Normal	243 (21.2)	1 [Reference]	NA
Abnormal	54 (42.7)	2.8 (1.6-4.8)	NA
**Total cholesterol^c^**			
Normal	237 (24.1)	1 [Reference]	NA
Abnormal	60 (41.7)	2.3 (1.3-4.1)	NA

Abbreviations: IR, insulin resistance; OR, odds ratio; CI, confidence interval; NA, not applicable;  HDL, high-density lipoprotein; LDL, low-density lipoprotein.

a Adolescent age categories were early adolescence, aged <15 y; middle adolescence, aged 15-17 y; and late adolescence, aged >17 y.

b The body mass index percentile categories of below-normal (<5th percentile) and normal (5th to <85th percentiles) have been combined.

c The normal values are HDL cholesterol >1.2 mmol/L for adolescent boys aged 14-19 y and >1.3 mmol/L for all others; LDL cholesterol <2.8 mmol/L; triglycerides <1.2 mmol/L; and total cholesterol <4.4 mmol/L (26,27).

## References

[1] Ogden CL, Carroll MD, Flegal KM (2008). High body mass index for age among US children and adolescents, 2003-2006. JAMA.

[2] Ogden CL, Carroll MD, McDowell MA, Flegal KM (2007). Obesity among adults in the United States — no statistically significant change since 2003-2004. NCHS Data Brief.

[3] Ogden CL, Carroll MD, Curtin LR, McDowell MA, Tabak CJ, Flegal KM (2006). Prevalence of overweight and obesity in the United States, 1999-2004. JAMA.

[4] Cook S, Gidding SS (2007). Modifying cardiovascular risk in adolescent obesity. Circulation.

[5] Hunt KJ, Lehman DM, Arya R, Fowler S, Leach RJ, Goring HH (2005). Genome-wide linkage analyses of type 2 diabetes in Mexican Americans: The San Antonio Family Diabetes/Gallbladder Study. Diabetes.

[6] Duncan GE (2006). Prevalence of diabetes and impaired fasting glucose levels among US adolescents: National Health and Nutrition Examination Survey, 1999-2002. Arch Pediatr Adolesc Med.

[7] Lawrence JM, Mayer-Davis EJ, Reynolds K, Beyer J, Pettitt DJ, D'Agostino RB (2009). Diabetes in Hispanic American youth: prevalence, incidence, demographics, and clinical characteristics: the SEARCH for Diabetes in Youth Study. Diabetes Care.

[8] (2002). Texas Pediatric Diabetes Research Advisory Committee. Pediatric diabetes research in Texas: an initiative to understand and prevent diabetes in Texas children.

[9] Bacha F, Saad R, Gungor N, Arslanian SA (2006). Are obesity-related metabolic risk factors modulated by the degree of insulin resistance in adolescents?. Diabetes Care.

[10] Goodarzi MO, Guo X, Taylor KD, Quiñones MJ, Saad MF, Yang H (2004). Lipoprotein lipase is a gene for insulin resistance in Mexican Americans. Diabetes.

[11] Singh-Manoux A, Adler NE, Marmot MG (2003). Subjective social status: its determinants and its association with measures of ill health in the Whitehall II study. Soc Sci Med.

[12] Bauman LJ, Silver EJ, Stein RE (2006). Cumulative social disadvantage and child health. Pediatrics.

[13] Franzini L, Fernandez-Esquer ME (2006). The association of subjective social status and health in low-income Mexican-origin individuals in Texas. Soc Sci Med.

[14] Miech RA, Kumanyika SK, Stettler N, Link BG, Phelan JC, Chang VW (2006). Trends in the association of poverty with overweight among US adolescents, 1971-2004. JAMA.

[15] Goodman E, Daniels SR, Dolan LM (2007). Socioeconomic disparities in insulin resistance: results from the Princeton School District study. Psychosom Med.

[16] US Census Bureau. American FactFinder.

[17] DeNavas-Walt C, Proctor BD, Lee CH (2006). US Census Bureau, current population reports, income, poverty, and health insurance coverage in the United States: 2005.

[18] (2001). Federal Reserve Bank of Dallas. The border economy.

[19] Brownsville Independent School District. Official website facts and statistics.

[20] Centers for Disease Control and Prevention. 2000 CDC growth charts.

[21] Fernandez JR, Redden DT, Pietrobelli A, Allison DB (2004). Waist circumference percentiles in nationally representative samples of African-American, European-American, and Mexican-American children and adolescents. J Pediatr.

[22] National High Blood Pressure Education Program Working Group on High Blood Pressure in Children and Adolescents (2004). The fourth report on the diagnosis, evaluation, and treatment of high blood pressure in children and adolescents. Pediatrics.

[23] Keskin M, Kurtoglu S, Kendirci M, Atabek ME, Yazici C (2005). Homeostasis model assessment is more reliable than the fasting glucose/insulin ratio and quantitative insulin sensitivity check index for assessing insulin resistance among obese children and adolescents. Pediatrics.

[24] Matthews DR, Hosker JP, Rudenski AS, Naylor BA, Treacher DF, Turner RC (1985). Homeostasis model assessment: insulin resistance and beta-cell function from fasting plasma glucose and insulin concentrations in man. Diabetologia.

[25] American Diabetes Association (2010). Standards of medical care in diabetes — 2010. Diabetes Care.

[26] de Ferranti SD, Gauvreau K, Ludwig DS, Neufeld EJ, Newburger JW, Rifai N (2004). Prevalence of the metabolic syndrome in American adolescents: findings from the Third National Health and Nutrition Examination Survey. Circulation.

[27] Grundy SM, Cleeman JI, Merz CN, Brewer HB, Clark LT, Hunninghake DB (2004). Implications of recent clinical trials for the National Cholesterol Education Program Adult Treatment Panel III guidelines. Circulation.

[28] Harrell JS, Jessup A, Greene N (2006). Changing our future: obesity and the metabolic syndrome in children and adolescents. J Cardiovasc Nurs.

[29] Lindstrom J, Peltonen M, Eriksson JG, Aunola S, Aunola S, Hämäläinen H (2008). Determinants for the effectiveness of lifestyle intervention in the Finnish Diabetes Prevention Study. Diabetes Care.

[30] Hirschler V, Aranda C, Calcagno Mde, Maccalini G, Jadzinsky M (2005). Can waist circumference identify children with the metabolic syndrome?. Arch Pediatr Adolesc Med.

[31] Huang TT, Nansel TR, Belsheim AR, Morrison JA (2008). Sensitivity, specificity, and predictive values of pediatric metabolic syndrome components in relation to adult metabolic syndrome: the Princeton LRC follow-up study. J Pediatr.

[32] Lee S, Bacha F, Gungor N, Arslanian S (2008). Comparison of different definitions of pediatric metabolic syndrome: relation to abdominal adiposity, insulin resistance, adiponectin, and inflammatory biomarkers. J Pediatr.

[33] Goodman E, Dolan LM, Morrison JA, Daniels SR (2005). Factor analysis of clustered cardiovascular risks in adolescence: obesity is the predominant correlate of risk among youth. Circulation.

[34] Lee JM, Gebremariam A, Card-Higginson P, Shaw JL, Thompson JW, Davis MM (2009). Poor performance of body mass index as a marker for hypercholesterolemia in children and adolescents. Arch Pediatr Adolesc Med.

